# The relationship between the emergency nurses' sleep quality and the sleep quality of their spouses: A cross‐sectional descriptive‐analytical study

**DOI:** 10.1002/hsr2.965

**Published:** 2022-11-29

**Authors:** Abbasali Ebrahimian, Ali Fakhr‐Movahedi, Seyed‐Hossein Hashemi‐Amrei

**Affiliations:** ^1^ Health in Emergencies and Disasters Group, Faculty of Paramedical Qom University of Medical Sciences Qom Iran; ^2^ Nursing Care Research Center Semnan University of Medical Sciences Semnan Iran; ^3^ Nursing School, Student Research Committee Semnan University of Medical Sciences Semnan Iran

**Keywords:** emergencies, nurses, sleep, spouses

## Abstract

**Background and Aims:**

Due to the heavy working shifts, emergency nurses may have to sleep at unusual times of the day, affecting their spouse's sleep. This study proposed to detect the relationship between the woman emergency nurse's sleep quality and the sleep quality of their spouses.

**Methods:**

This cross‐sectional descriptive‐analytical study has lasted for 4 months since June 21, 2020. The study population was all women nurses working at a hospital emergency department. The data was collected by a demographic questionnaire and Pittsburgh sleep quality index (PSQI) for nurses and their spouses. The data were analyzed by descriptive and inferential statistics.

**Results:**

The prevalence of sleep quality disorder among female emergency department nurses and their husbands was 82.7% and 80.6%, respectively. The mean sleep quality score of female nurses and their husbands was 8.46 ± 4.43 and 6.50 ± 2.52, respectively. A strong and positive correlation was found between the PSQI score of female nurses and their husbands (*p* < 0.001). The regression model showed that increasing the body mass index (BMI) of female emergency nurses can decrease their sleep quality. However, increasing the BMI of female emergency nurses' spouses and their work experience in the emergency department can improve their sleep quality.

**Conclusion:**

The sleep quality of female emergency department nurses was directly correlated with their husbands' sleep quality. Therefore, the sleep quality of nurses working in the emergency departments and their spouses should be examined periodically.

## 1 INTRODUCTION

Many nurses who work rotating shifts have irregular sleep patterns.^1^ In studies performed by Zhi‐hao Tu et al., Bazrafshan et al., and Xuexue Deng et al., nearly 60%, 56%, and 46% of nurses had low sleep quality, respectively.^2^, ^3^, ^4^ The low sleep quality of nurses negatively affects their health and reduces their performance during patient care.^1^, ^2^, ^3^, ^4^, ^5^ It can also cause stress, depression, fatigue, burnout, obesity, cardiovascular disorders, and mental disorders.^6^, ^7^, ^8^, ^9^

Nurses working in emergency departments have lower sleep quality due to constant contact with critical patients. Studies conducted by Dong et al., Suleiman et al., and Weaver et al. reported that the prevalence of sleep quality disorders in emergency nurses is 65.8%, 92.1%, and 73.3%, respectively.^10^, ^11^, ^12^ Reduced sleep quality is tied to fatigue, sleep, cognitive disorders, mental disorders, increased likelihood of medical errors, and declined job satisfaction in emergency nurses.^12^, ^13^, ^14^, ^15^ Decreased sleep quality and its subsequent problems can also affect the married lives of nurses, including decreased sleep quality of their spouses.

Nurses tend to sleep both before and after night shifts,^16^ which may influence the sleep of their spouses. Walters et al.^17^ reported that synchronized sleep patterns of couples could promote their psychosocial health. Tracy et al.^18^ also argued a direct association between couples' sleep quality. Lee et al.^19^ showed a positive correlation between sleep duration and couples' sleep quality. Besides, this study mentioned the wife's sleep duration as a predictor of a man's sleep duration. Heather et al.^20^ also reported that synchronized sleep patterns of couples could reduce blood pressure and decrease CRP. Liu et al.^21^ and Zhang et al.^22^ showed that poor sleep quality could cause work‐family conflict in nurses. In reality, the sleep pattern of nurses is different from their spouses, which may cause physical and/or mental problems and reduce their quality of life.^22^ Considering that the sleep quality of emergency nurses is lower than their counterparts, it seems that their spouses also have lower sleep quality than the spouses of other nurses. The current study aimed to investigate the association between the women emergency nurse's sleep quality and the sleep quality of their spouses.

## 2 METHODS

### 2.1 Study design and participants

This cross‐sectional descriptive‐analytical study has lasted for 4 months since June 21, 2020. The study population was all female nurses working at a 25 Mazandaran university hospital emergency department and had the inclusion criteria.

#### 2.1.1 Inclusion criteria

The participant and his spouse are willing to participate in the research. They must have completed the informed consent form before starting the study. When completing the questionnaires, the female nurses should have at least 1 year of working experience in the hospital's emergency department. They should not have the habit of using any sleeping pills or drinks to sleep or stay awake. When completing the questionnaire, they should not have pain or other physical and mental problems. They must be married, their husbands must not be nurses, and they must not have any restrictions on sexual activity with their husbands. Also, they do not have any history of previously known sleep disorders.

#### 2.1.2 Exclusion criteria

Unwillingness to continue participating in the study and, failure to complete and send the questionnaire.

### 2.2 Sample size

The sample size was estimated by using the PASS 11, version 11.0.8. At first, a pilot study was performed on the 15 nurses with their husbands. Then according to the correlation coefficient between sleep scores of nurses with their husbands (*R* = 0.626, *p* = 0.01), a significance level (*α*) of 0.01, and power of 0.99 using the test for two correlation, a sample size of 93 nurses with their husbands (total 186 samples) was calculated. To prevent a dropout in the sample size, it was increased to 117 nurses with their husbands (total 223) subjects by a 20% dropout rate.

### 2.3 Measures

The data collection tools were a demographic questionnaire for the nurse, a demographic questionnaire for the nurse's spouses, and Pittsburgh sleep quality index (PSQI). This questionnaire was completed after receiving informed consent.

### 2.4 Demographic questionnaire

This questionnaire includes age, gender, body mass index (BMI), number of children, duration of the marriage, work experience, number of shifts per month, number of night shifts per month, duration of employment in the emergency department, and enough sleep after a night shift.

In Iran, the night shift of nurses is 12 h and lasts from 8 p.m. to 8 a.m. During these 12 h, they usually do not sleep but may rest if they do not have special duties. Most of them go to their private homes after working at night and sleep there. In this study, we asked them if the amount of sleep they get after a night shift is enough? Usually, night shift nurses eat breakfast in the hospital. Hospital breakfast is usually bread, cheese, butter, eggs, milk, and tea. After eating breakfast, they would go to their private homes and sleep. Therefore, their diet before sleep was almost the same. The sleeping environment has also been their home.

### 2.5 PSQI

The PSQI assesses the sleep quality and disturbance retrospectively over 1 month. This is a 19 item self‐report scale that measures sleep quality, and the survey can be completed within 5 min. This questionnaire is scored on a 4‐point Likert scale from 0 to 3 (0, not in the past month; 1, less than once per week; 2, once or twice per week; and 3, three or more times per week) the total score ranges from 0 to 21, with higher scores indicating poorer sleep quality. This questionnaire has seven subscales: Subjective sleep quality, sleep latency, sleep duration, habitual sleep efficiency, sleep disturbance, sleeping medication use, and daytime dysfunction.^23^ The Persian version of the PSQI‐P was used in the present study. Cronbach's *α* coefficient for all subjects was 0.77. Sensitivity and specificity are high (94% and 72%, respectively) for a PSQI‐P cutoff value of 5.^24^

### 2.6 Procedures

A questionnaire on demographic information and a sleep quality questionnaire was used to collect the data. Participating nurses were asked to give the questionnaires to their spouses. Written informed consent was obtained from all nurses and their spouses willing to participate in the study. Then, the researcher provided the participants with two stamped envelopes with a return address (but no sender's address) and asked them to put the envelope in the nearest mailbox after completion. No code or mark was placed on the questionnaires to observe the confidentiality of information. However, they were asked to write a common code on both questionnaires to facilitate the identification of questionnaires or put both questionnaires in one envelope. Also, those participants who were willing to know their sleep quality status were asked to write their names and contact numbers on the questionnaire.

### 2.7 Statistical methods

All analyses were performed using SPSS‐16 software (SPSS Inc.). The Kolmogorov−Smirnov test showed that the data distribution was normal. The obtained data were analyzed using descriptive statistics, including frequency, mean, and standard deviation. The Pearson's correlation test was used to demonstrate the relationship between the participant's age, number of children, duration of the marriage, work experience, number of shifts during the month, number of night shifts during the month, and work experience in the emergency department with PSQI scores. The Spearman's correlation test demonstrated the relationship between the participants' daily sleep status after night work with PSQI scores. An independent samples *t*‐test was used to compare the mean PSQI scores in female emergency nurses and their spouses. The level of statistical significance was set at *p* < 0.05 for all statistical analyses.

Multiple linear regression analysis to test predictability between the dependent variable of sleep quality of female nurses working in the emergency department with the independent variables of sleep quality, age, BMI, number of children, duration of marriage, amount of clinical work experience, number of night shifts, amount of hours overtime per month, the duration of work in the emergency department in female nurses, and the age and BMI of their husbands were done.

## 3 RESULTS

Initially, 106 nurses working in the emergency department and their spouses accepted to participate in the study, and 8 (7.54%) of them were excluded due to various reasons such as the incompleteness of questionnaires (5 subjects) and unwillingness to continue the study (2 subjects). Therefore, data of 98 couples (196 subjects) were analyzed.

The mean age of female emergency departments' nurses and their spouses was 33.66 ± 5.74 and 37.16 ± 5.82, respectively. The mean BMI of female nurses and their spouses was 25.69 ± 3.17 and 25.86 ± 2.86, respectively. There was no significant difference between the two groups (*p* > 0.05). All participants were living with their first husbands, and none of the participants had remarried. The mean duration of participants' marriage was 10.35 ± 5.28. The mean female nurses' work experience was 9.84 ± 4.66.

The mean number of female nurses' monthly and night shifts was 27.64 ± 2.80 and 6.82 ± 2.16 shifts, respectively. The mean female nurses' work experience in the emergency department was 5.91 ± 3.16 years (Table 1).

**Table 1:** Participants' demographic variables in the two groups

Variables		Female nurses *n* (%)	Spouses of female nurses *n* (%)
Age (year)	Young (18−35)	63 (64.3)	39 (39.8)
	Middle‐aged (36−45)	33 (33.7)	50 (51)
	Adult (46−65)	2 (2)	9 (9.2)
	Mean ± SD	33.66 ± 5.74	37.16 ± 5.82
BMI	Under‐weight (<18.5)	2 (2)	0 (0)
	Normal (18.5−24.9)	38 (38.8)	49 (50)
	Over‐weight (25−29.9)	48 (49)	42 (42.9)
	Obese (>30)	10 (10.2)	7 (7.1)
	Mean ± SD	25.69 ± 3.17	25.86 ± 2.86
Child number	0	13 (13.3)	13 (13.3)
	1	54 (55.1)	54 (55.1)
	2	30 (30.6)	30 (30.6)
	3	1 (1)	1 (1)
	4	0 (0)	0 (0)
	Mean ± SD	1.19 ± 0.66	1.19 ± 0.66
Duration of marriage (year)	1−10	59 (60.2)	59 (60.2)
	11−20	38 (38.8)	38 (38.8)
	>20	1 (1)	1 (1)
	Mean ± SD	10.35 ± 5.28	10.35 ± 5.28
Working experience (year)	0	0 (0)	0 (0)
	1−10	56 (57.2)	80 (81.7)
	11−20	39 (39.8)	15 (15.2)
	>20	3 (3)	3 (3.1)
	Mean ± SD	9.84 ± 4.66	12.85 ± 4.80
Working experience in the ED (year)	1−10	44 (80)	—
	11−20	11 (20)	—
	>20	0 (0)	—
	Mean ± SD	5.91 ± 3.16	—
Number of monthly shifts	15−20	3 (3)	—
	21−25	11 (11.2)	—
	26−30	76 (77.6)	—
	>30	8 (8.2)	—
	Mean ± SD	27.64 ± 2.80	—
Number of monthly night shifts	<5	27 (27.6)	—
	6−10	65 (66.3)	—
	>10	6 (6.1)	—
	Mean ± SD	6.82 ± 2.16	—
enough sleep after a night shift	Always	6 (6.1)	—
	Mostly	64 (65.3)	—
	Sometimes	25 (25.5)	—
	Seldom	3 (3.1)	—

Abbreviation: BMI, body mass index.

The Pearson's correlation coefficient showed a significant correlation between sleep quality scores of female nurses and the duration of the marriage (*p* = 0.046), and the number of shifts in each month (*p* = 0.047). There was no correlation between sleep quality scores of female nurses and age (*p* = 0.097), BMI (*p* = 0.190), number of children (*p* = 0.094), work experience (*p* = 0.060), number of night shifts in each month (*p* = 0.143), and work experience in the emergency department (*p* = 0.176) (Table 2).

**Table 2:** Correlation between female nurse demographic data and PSQI scores

Variables	Mean ± SD	*p* Value^a^
Age	33.66 ± 5.74	0.097
PSQI score	8.46 ± 4.43	
BMI	25.69 ± 3.17	0.190
PSQI score	8.46 ± 4.43	
Child number	1.19 ± 0.66	0.094
PSQI score	8.46 ± 4.43	
Duration of marriage (year)	10.35 ± 5.28	0.046
PSQI score	8.46 ± 4.43	
Working experience (year)	9.84 ± 4.66	0.060
PSQI score	8.46 ± 4.43	
Working experience in the ED (year)	5.91 ± 3.16	0.176
PSQI score	8.46 ± 4.43	
Number of monthly shifts	27.64 ± 2.80	0.047
PSQI score	8.46 ± 4.43	
Number of monthly night shifts	6.82 ± 2.16	0.143
PSQI score	8.46 ± 4.43	

Abbreviations: BMI, body mass index; PSQI, Pittsburgh sleep quality index.

^a^ Based on Pearson's correlation coefficient.

The prevalence of sleep quality disorder among female nurses and their husbands was 82.7% and 80.6%, respectively. The mean sleep quality score of female nurses and their husbands was 8.46 ± 4.43 and 6.50 ± 2.52, respectively. A strong and positive correlation was found between the PSQI score of female nurses and their husbands (*p* < 0.001). There was a positive correlation between sleep quality scores of female nurses and their husbands in subscales of subjective sleep quality (*p* = 0.048), sleep duration (*p* = 0.020), and daytime dysfunction (*p* = 0.013) (Table 3).

**Table 3:** Correlation between PSQI subscale scores in the female emergency nurses with their spouses

PSQI component		Female nurses	Spouses of female nurses
Subjective sleep quality	Mean ± SD	1.36 ± 0.77	0.23 ± 0.45
	*p* Value^a^	0.048	
Sleep latency	Mean ± SD	1.54 ± 0.77	1.14 ± 0.67
	*p* Value^a^	0.490	
Sleep duration	Mean ± SD	1.32 ± 1.10	1.15 ± 0.84
	*p* Value^a^	0.020	
Sleep efficiency	Mean ± SD	0.46 ± 0.83	0.15 ± 0.84
	*p* Value^a^	0.502	
Sleep disturbance	Mean ± SD	1.22 ± 0.52	1.02 ± 0.43
	*p* Value^a^	0.113	
Use of sleep medication	Mean ± SD	0.30 ± 0.66	0.08 ± 0.31
	*p* Value^a^	0.502	
Daytime dysfunction	Mean ± SD	2.25 ± 1.89	1.59 ± 1.36
	*p* Value^a^	0.013	
Total PSQI scores	<5 *n* (%)	17 (17.3)	19 (19.4)
	>5 *n* (%)	81 (82.7)	79 (80.6)
	Mean ± SD	8.46 ± 4.43	6.50 ± 2.52
	*p* Value^a^	**≤0.001**	

*Note*: Bold value indicates statistically significant *p* < 0.05.

Abbreviation: PSQI, Pittsburgh sleep quality index.

^a^ Based on Pearson's correlation coefficient.

Regression analysis showed that the independent variables of BMI of female nurses, BMI of their husbands, and duration of work in the emergency department significantly determined the quality of sleep of female nurses working in the emergency department. The fitting regression model was as follows:

Total sleep scores of nurses = 8.678 + (0.462 × [BMI of nurses]) – (0.388 × [BMI of nurses' husbands]) – (0.343 × [working experience in the ED]).

In the above model, work experience in the emergency department and the BMI of spouses have a negative coefficient. The total nurses' sleep quality score decreased for each unit increase in these variables. Since a higher score of the Pittsburgh index indicates worse sleep quality, in fact, with the increase of husbands' BMI variables and the duration of work in the emergency department, the nurses' sleep quality improved (Tables 4 and 5).

**Table 4:** ANOVA, summary model, regression, and adjusted regression coefficients for total sleep score^a^ in female nurses

Model	*R*	*R* ^2^	Adjusted *R* ^2^	Sum of squares		*F*	*p* Value
				Regression	Residual		
1	0.204^b^	0.042	0.032	79.421	1826.987	4.173	0.044
2	0.321^c^	0.103	0.084	197.041	1709.367	5.475	0.006
3	0.397^d^	0.157	0.130	299.720	1606.689	5.845	0.001

Abbreviation: BMI, body mass index.

^a^ Depended variable: Total sleep score of nurses.

^b^ Predictors: constant, BMI of nurses' husbands.

^c^ Predictors: constant, BMI of nurses' husbands, and BMI of nurses.

^d^ Predictors: constant, BMI of nurses' husbands, BMI of nurses, and time of working in an emergency setting.

**Table 5:** Regression coefficient of predictive variables (independent variables)

Model		Unstandardized coefficients		Standardized coefficients beta	*t*	*p* Value
		*B*	Standard error			
1	Constant	16.649	4.028		4.133	<0.001
	BMI of nurses' husbands	−0.316	0.155	−0.204	−2.043	0.044
2	Constant	10.011	4.699		2.130	0.036
	BMI of nurses' husbands	−0.415	0.155	−0.268	−2.671	0.009
	BMI of nurses	0.358	0.140	0.256	2.557	0.012
3	Constant	8.678	4.612		1.882	0.063
	BMI of nurses' husbands	−0.388	0.152	−0.250	−2.555	0.012
	BMI of nurses	0.462	0.143	0.331	3.232	0.002
	Time of work in an emergency	−0.343	0.141	−0.246	−2.451	0.016

*Note*: Dependent variable: Total sleep scores of nurses.

Abbreviation: BMI, body mass index.

## 4 DISCUSSION

This study examined the relationship between female emergency nurses' sleep quality and the sleep quality of their spouses. The prevalence of sleep quality disorder among female nurses working in the emergency department was 82.7%. Suleiman et al.^11^ reported a prevalence of 92.1% for emergency nurses' sleep disorders, which was very close to the current study. However, several studies reported lower sleep qualities for nurses working in emergency departments. For example, studies conducted by Dong et al.,^10^ Weaver et al.,^12^ and Guo and Wang et al.^25^ reported a prevalence of 65.8%, 73.3%, and 63.40%, respectively, for sleep disorders. The higher prevalence of sleep disorders in the present study is due to different occupational conditions of Iranian nurses working in emergency departments compared to other countries; mainly, the shortage of health staff in Iran has caused an increased workload of nurses. Also, because of noncompliance with standards on nurse‐to‐patient ratio, Iranian emergency nurses have a higher workload than their counterparts in developing countries.^26^, ^27^

The total mean score of PSQI in female emergency nurses was 8.46 ± 4.43. This means in the study of Dong et al.^10^ was 8.2 ± 3.9 and in the study of Weaver et al.^12^ was 7.3 ± 3.6, which was close to the results of the present study. This finding shows that emergency nurses have sleep disorders in many countries worldwide. Therefore, appropriate actions should be taken to correct their sleep patterns.

Factors such as duration of the marriage and the number of shifts in each month had a significant correlation with the sleep quality of female emergency nurses. Dong et al.^10^ also reported that female emergency nurses have lower sleep quality than their male counterparts. They also showed that the number of night's shifts per month could reduce the sleep quality of nurses. The low number of factors associated with reduced sleep quality in female nurses indicates that the high impact of working in the emergency department has faded the impact of other variables. Therefore, it is suggested that married female nurses not be used as much as possible in the emergency department. The mean PSQI score of female nurses was 8.16, while Dong et al.^10^ and Weaver et al.^12^ reported a mean score of 8.2 and 7.3, respectively. This finding indicates that nurses working in emergency departments suffer from sleep disorders in many countries. Therefore, appropriate measures are needed to correct their sleep patterns.

There was a significant difference between the mean PSQI scores of female nurses and their husbands. So husbands of female emergency nurses' departments were suffering from sleep disorders. Some studies reported a direct association between couples' sleep quality.^17^, ^18^ After finishing a working shift, most nurses experience fatigue and usually sleep. Their shifts are often irregular; hence their sleep pattern is also irregular. However, their spouses tend to have a regular sleep pattern. However, their sleep pattern will also be impaired over time because when they need to sleep, their spouses (nurses) do not need to sleep, and vice versa. This problem is more severe during night sleep.

Nurses with rotating shifts usually get used to being wakeful and do many of their activities, such as talking to their spouse, watching TV, listening to music, cooking, washing clothes and dishes, reading books, and so forth, in the final hours of the night. Therefore, the home environment is not appropriate for their spouses to sleep in; hence they will experience sleep problems. Therefore, measures are needed to improve their sleep quality. Increasing the subjective feelings of emergency nurses may be the most critical reason or mechanism to analyze the relationship between emergency nurses' sleep quality and their spouses' sleep quality.

Because as the findings of the present study showed, nurses working in emergency departments and their spouses suffer from dysfunctions in their daily activities. To the best knowledge of the authors, no study has investigated the sleep quality of children of nurses with rotating work shifts, but probably the decreased sleep quality of nurses and their spouses, in turn, reduces the sleep quality of their children. Therefore, it is suggested to use single nurses in the emergency departments. Moreover, by scheduling fixed working shifts, the sleep pattern of nurses working in emergency departments would be corrected.

The regression model showed that increasing the BMI of female emergency nurses can decrease their sleep quality. However, increasing the BMI of female emergency nurses’ spouses and their work experience in the emergency department can improve their sleep quality. Miguez‐Torres et al.^28^ showed in a study that nurses who have worked for several years showed a better ability to regulate emotions than those with less experience. Those who were overweight in grade II and obese in type I expressed their feelings better. Also, the regulation of emotional states decreased as weight increased. Chang and Yang^29^ showed in a study that the BMI of female nurses can affect their sleep quality. It seems that nurses with a higher BMI are more tired when working in the emergency department than those with a lower BMI. So they need more sleep but cannot sleep enough after going to someone's house. Because of this, the quality of their sleep decreases. The bodies of nurses with more work experience in the emergency department are more compatible with insomnia caused by night shift work. They also learn what mechanisms to use to reduce fatigue while working in the emergency department. They also learn when is the best time for them to sleep. Therefore, increasing the work experience in the emergency department can improve the sleep quality of nurses.

### 4.1 Study limitations

We asked participants to complete the questionnaires without the assistance and advice of their spouses or others. However, some of them may not have followed our advice. Therefore, this study is limited in this regard.

## 5 CONCLUSION

The prevalence of female emergency nurses' sleep quality and their husbands was high. The sleep quality of female nurses was associated with their husbands. There was a significant relationship between the female emergency nurses' sleep quality with the duration of the marriage and the number of shifts each month. Also, increasing the BMI of female emergency nurses can decrease their sleep quality. However, increasing the BMI of female emergency nurses' spouses and their work experience in the emergency department can improve their sleep quality. Therefore, it is recommended that married female nurses with a high BMI are not used in the emergency room. The working environment of female emergency room nurses with a high work experience in this department should not be changed. Also, the quality of sleep of nurses working in the emergency room and their spouses should be examined periodically to provide the necessary information to make relevant decisions. More studies are needed to investigate the sleep quality of emergency nurses and provide appropriate solutions to increase their sleep quality.

## AUTHOR CONTRIBUTIONS

**Abbasali Ebrahimian**: conceptualization; methodology; project administration; resources; supervision; validation; writing – original draft; writing – review & editing. **Ali Fakhr‐Movahedi**: conceptualization; formal analysis; methodology; validation; writing – original draft; writing – review & editing. **Seyed‐Hossein Hashemi‐Amrei**: conceptualization; data curation; investigation; methodology; writing – review & editing. All authors have read and approved the final version of the manuscript.

## CONFLICT OF INTEREST

The authors declare no conflict of interest.

### ETHICS STATEMENT

The Ethics Committee of Semnan University of Medical Sciences approved this study protocol (IR.SEMUMS.REC.1398.025). Before initiating the study, the researchers provided the Ethical Committee approval to the hospital authorities affiliated with the Mazandaran University of Medical Sciences. All emergency nurses and their spouses were informed about the study's objectives and how to complete the questionnaires. If a nurse or her/his spouse were not willing to continue the study, they were excluded. Also, informed written consent was obtained from all participants.

## TRANSPARENCY STATEMENT

The lead author Seyed‐Hossein Hashemi‐Amrei affirms that this manuscript is an honest, accurate, and transparent account of the study being reported; that no important aspects of the study have been omitted; and that any discrepancies from the study as planned (and, if relevant, registered) have been explained.

## Data Availability

The data that support the findings of this study are available on request from the corresponding author. The authors confirm that the data supporting the findings of this study are available within the article. The lead author Mr. Seyed‐Hossein Hashemi‐Amrei has full access to all of the data in this study and takes complete responsibility for the integrity of the data and the accuracy of the data analysis.
